# Critical Involvement of CD44 in T Helper Type 2 Cell-Mediated Eosinophilic Airway Inflammation in a Mouse Model of Acute Asthma

**DOI:** 10.3389/fimmu.2021.811600

**Published:** 2022-01-07

**Authors:** Shigeki Katoh

**Affiliations:** Department of Respiratory Medicine, Kawasaki Medical School, Okayama, Japan

**Keywords:** acute asthma, CD44, CD44-deficient mice, hyaluronan, Th2 cell, Neu1 sialidase

## Abstract

Interactions between CD44 and hyaluronan (HA) are crucial for recruiting leukocytes to inflamed tissues. This review summarizes findings from our studies of the roles of CD44-HA interactions in leukocyte trafficking, with a particular focus on airway T helper type 2 (Th2) cells in mouse models of acute asthma. In a mite allergen-induced model of acute asthma, intraperitoneal injection of anti-CD44 monoclonal antibodies blocked lymphocytes and eosinophils from accumulating in the lung, and suppressed both the antigen-induced increase in Th2 cytokines in the bronchoalveolar lavage fluid (BALF) and airway hyperresponsiveness (AHR). CD44 deficiency was associated with decreased mite allergen-induced Th2 cell-mediated airway inflammation and AHR in sensitized mice. Asthmatic responses to antigen-sensitized splenic CD4^+^ T cells transferred from CD44-deficient mice were weaker than in wild-type mice. Administration of anti-CD44 monoclonal antibodies preferentially suppressed the airway accumulation of antigen-specific Th2 cells induced by antigen challenge, without affecting Th1 and Th17 cells. Increased HA-binding ability of CD44 and expression of Neu1 sialidase were observed on antigen-specific Th2 cells compared with antigen-specific Th1 and Th17 cells. Finally, in a mouse model of acute asthma, neuraminidase 1-deficient SM/J mice exhibited a lower Th2 cytokine concentration and a lower absolute Th2 cell number in the BALF, as well as an attenuated AHR. Our findings indicate that CD44 critically contributes to the antigen challenge-induced airway accumulation of antigen-specific Th2 cells, without affecting Th1 and Th17 cells, in mice. Furthermore, neuraminidase 1 activity is necessary for the interaction between HA and CD44, and Th2 cell-mediated airway inflammation.

## Introduction

The cell surface adhesion receptor cluster of differentiation 44 (CD44) is a heavily glycosylated molecule that regulates the adhesion of lymphocytes to inflamed endothelial cells, T cell activation, tumor metastasis, and many other cellular processes. While hyaluronan (HA) is the principal ligand of CD44, only a few types of cells use CD44 to recognize HA (1, 2). The structural variability of CD44 might affect its ability to recognize HA. Sialic acid is a terminal sugar chain of glycoproteins followed by a β-galactoside, such as CD44, that is catalyzed by neuraminidase. The 4 known mammalian neuraminidases (Neu1, Neu2, Neu3, and Neu4) are involved in variety of physiological processes (3). We demonstrated that CD44 glycosylation, such as by sialic acid, negatively regulates its recognition of HA (4).

Both CD44 and HA critically contribute to leukocyte recruitment to many organs *in vivo* (5). Disease severity and recruitment of lymphocytes in animal models of acute asthma, arthritis, and graft-versus-host disease are reduced by antibody blockade of CD44 or CD44 deficiency, as well as by enzymatic depletion of endothelial HA (6–8). In addition to the support function of CD44-HA interactions in lymphocyte rolling, direct association between CD44 and integrins enabling high-affinity binding to vascular cell adhesion molecule 1 is necessary for lymphocyte adhesion (9, 10).

**Figure 1:**
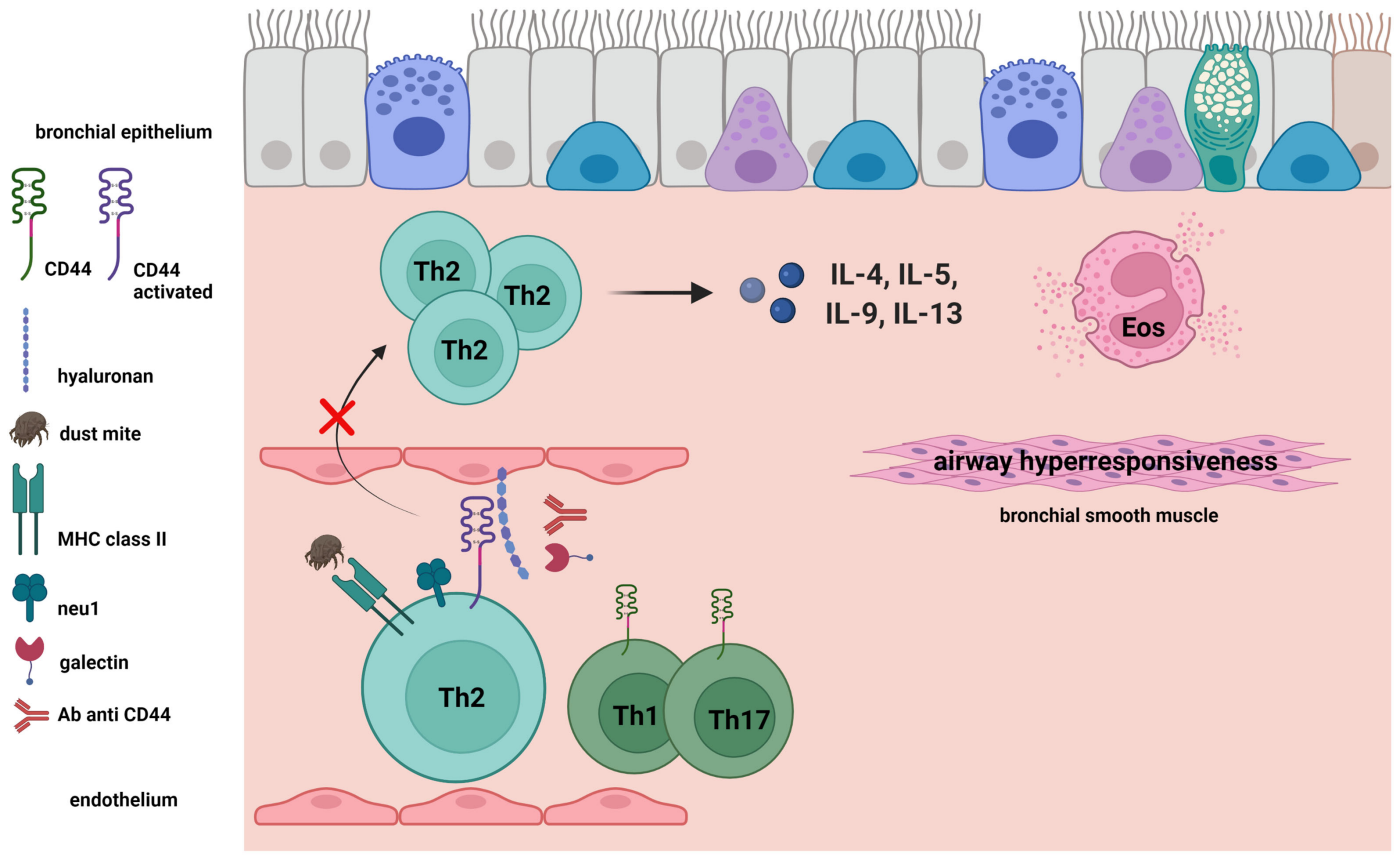
Antigen-induced activation of CD44 expressed on Th2 cells. CD44 receptor activity for hyaluronan (HA) and expression of neuraminidase 1 (Neu1) on Th2 cells were induced by antigen challenge in a mouse model of acute asthma. We demonstrated the importance of Th2 cell recruitment in the bronchus by CD44-HA interactions in the pathogenesis of acute allergic asthma in an experimental mouse model. Galectin-9 and anti-CD44 mAb inhibit the CD44-HA interaction, as well as the following Th2-mediated eosinophilic airway inflammation and airway hyperresponsiveness.

Asthma is a condition that presents with reversible airway obstruction, chronic airway inflammation, features of bronchial remodeling, and airway hyperresponsiveness (AHR) (11), and is considered to develop in response to the airway accumulation of antigen-activated CD4^+^ T cells (12). We investigated how CD44 participates in the airway accumulation of CD4^+^ T cells by developing an asthmatic phenotype in a mouse model of allergic acute asthma. In this review, we discuss how CD44-HA interactions are involved in CD4^+^ T cell trafficking. We also explore the mechanisms regulating these interactions, and highlight the importance of T helper type 2 (Th2) cell recruitment by CD44-HA interactions in the pathogenesis of acute allergic asthma in an experimental mouse model (**Figure 1**).

## CD44 Glycosylation Negatively Affects Its Recognition of HA

CD44 is a heavily glycosylated cell adhesion molecule deriving from alternative splicing of a single gene and modifications of the emerging protein. Lymphocytes are the best known cell type that uses CD44 to recognize and bind its ligand HA through their activation (13, 14). We demonstrated that protein glycosylation disruption in some cell types may increase their HA recognition. Flow cytometry to analyze the HA-binding ability of CD44 alone was performed using a purified CD44-immunoglobulin fusion protein and fluorescein-conjugated HA. Enhanced HA binding ability was observed when the CD44 fusion protein was treated with neuraminidase (4). These findings indicate that modifications of carbohydrates in CD44 may prevent its recognition of abundant HA in the body.

## Role of CD44 in Allergen-Induced Acute Airway Inflammation in Mice

The immunopathology of allergic respiratory inflammation may be due in part to the airway accumulation of CD4^+^ T cells and eosinophils following antigen activation (12). When an antigen is administered in a mouse model of acute asthma, the lungs begin to accumulate CD44-highly expressing CD4^+^ T cells (15). Therefore, we investigated the contribution of CD44 to allergen-induced acute respiratory inflammation in a mouse model of allergic asthma induced by intranasal administration of Ascaris sum extract and mite antigens followed by treatment with 2 anti-CD44 monoclonal antibodies (mAbs), and analyzed the bronchoalveolar lavage fluid (BALF) contents and AHR. The mAb KM201, directly prevents CD44-HA binding (16), whereas the mAb IM7 promotes receptor shedding from the cell surface (17). Injection of anti-CD44 antibodies to prevent CD44-HA binding abolished eosinophil and lymphocyte infiltration into the airways and reduced Th2 cytokine, interleukin (IL)-4, and IL-5 levels. Anti-CD44 treatment, however reduced the allergen-induced AHR. These findings suggest that CD44 is critically involved in the progression of acute allergic respiratory inflammation (6).

## Galectin-9 Inhibits CD44-HA Interactions and Reduces Symptoms in a Mouse Model of Acute Asthma

Galectin-9 (Gal-9) is a β-galactoside-binding protein that has roles in cell adhesion, chemoattraction, activation, and apoptosis (18). Hirashima et al. observed that Gal-9 induces the apoptosis of activated T cells in humans (19). Zhu et al. revealed that Gal-9 promotes Th1, but not in Th2, cell death in mice *via* a Tim-3–dependent pathway (20). We unexpectedly found that Gal-9 directly binds CD44, which blocks the CD44-HA interaction. To investigate the involvement of Gal-9 in the pathogenesis of allergic airway inflammation, we administered stable human Gal-9 (21) in a mouse model of acute asthma induced by intranasal administration of mite allergen. Intravenous injection of Gal-9 reduced both AHR and Th2 cell-associated airway inflammation induced by the mite allergen in sensitized mice. In addition, administration of Gal-9 impeded the airway infiltration of peripheral blood Th2 cells (22). Taken together, these findings indicate that Gal-9 inhibits allergen-induced airway inflammation and AHR by regulating the CD44-mediated leukocyte recognition of HA.

## CD44 Is Critical for the Airway Accumulation of Antigen-Specific Th2 Cells, Following Antigen Challenge in Mice

We studied the contribution of CD44 expressed on CD4^+^ T cells to the airway accumulation of Th2 cells using CD44-deficient mice and anti-CD44 mAbs. The CD44-deficency was associated with decreased mite allergen-induced Th2 cell-mediated airway inflammation in sensitized mice. Asthmatic responses to antigen-sensitized splenic CD4^+^ T cells transferred from CD44-deficient mice were weaker than in wild-type mice. We then assessed CD44 receptor activity for HA and expression of Neu1 sialidase on ovalbumin (OVA)-specific Th1, Th2, and Th17 cells *in vitro*, as previously described (23). OVA-specific Th2 cells more highly expressed Neu1 sialidase and exhibited higher CD44 HA receptor activity than OVA-specific Th1 and Th17 cells. Anti-CD44 mAbs preferentially suppressed the antigen challenge-induced accumulation of these Th2 cells in the airway, as compared with Th1 and Th17 cells in a mouse Th cell-transfer model (24, 25). Together, these findings demonstrated that CD44-expressing CD4^+^ T cells are critical for the airway accumulation of antigen-specific Th2 cells, but not Th1 or Th17 cells (**Figure 1**).

## Neu1 Sialidase Has a Crucial Role in the HA Receptor Function of CD44 in Th2 Cell-Mediated Airway Inflammation in an Acute Asthma Mouse Model

Sialic acid residues in CD44 negatively regulate the function of CD44, and CD44 is critically involved in the airway accumulation of Th2 cells in a mouse model of acute asthma (15, 24, 25). We therefore investigated how sialidase is involved in CD44-HA interactions on CD4^+^ T cells, and how it contributes to mite allergen-induced acute asthma in a mouse model. In splenic CD4^+^ T cells obtained from the model mice, the HA receptor activity of CD44 and Neu1 sialidase expression were increased after culture with the antigen. The antigen-induced HA binding ability of CD44 was markedly suppressed by a sialidase inhibitor. Binding of HA to CD44, however, was not observed in Neu1-deficient SM/J mice with a partial deficiency of lysosomal sialidase (26, 27). Further, the Neu1-deficient SM/J mice also exhibited a lower Th2 cytokine concentration and a lower absolute Th2 cell number in the BALF (27). These findings together indicate that Neu1 sialidase is required for the CD44-HA interaction and the development of acute asthmatic inflammation. It may be that enzyme activity remodels the cell surface CD44 expressed on CD4^+^ T cells, thereby altering the ability of CD44 to interact with HA.

## Conclusion

CD44-HA interactions critically contribute to the airway accumulation of allergen-specific Th2 cells in allergen-induced acute asthma mouse models. Neu1 sialidase activity in Th2 cells is a mechanism of CD44 receptor activation for binding HA (**Figure 1**). These findings suggest that CD44 and Neu1 sialidase could be candidate treatment targets for Th2 cell-mediated acute airway inflammation. Additional studies are needed to clarify the detailed role of CD44 in the development of chronic airway inflammation, such as human asthma, and to clarify the possible involvement of CD44 in other immune cell mechanisms underlying asthma pathophysiology.

## Author Contributions

The author confirms being the sole contributor of this manuscript and approved it for publication.

## Funding

This work was supported in part by Core Research for Evolutional Science and Technology of the Japan Science and Technology Agency and Grants-in-Aid for Scientific Research, Japan.

## Conflict of Interest

The author declares that the research was conducted in the absence of any commercial or financial relationships that could be construed as a potential conflict of interest.

## Publisher’s Note

All claims expressed in this article are solely those of the authors and do not necessarily represent those of their affiliated organizations, or those of the publisher, the editors and the reviewers. Any product that may be evaluated in this article, or claim that may be made by its manufacturer, is not guaranteed or endorsed by the publisher.
